# Implementing the national strategy of salt reduction in Morocco: the baker´s perspective

**DOI:** 10.11604/pamj.2020.37.337.27139

**Published:** 2020-12-11

**Authors:** Meryem Bouhamida, Nada Benajiba, Yasmine Guennoun, Sara Ait Lachguer, Nour Eddine Elhaloui, Fatima-Ezzahra Zahrou, Samir Mounach, Khalid El Kari, Ayoub Al-Jawaldeh, Amina Barkat, Hasnae Benkirane, Hassan Aguenaou

**Affiliations:** 1 Joint Research Unit in Nutrition and Food, Regional Designated Center of Nutrition Associated with International Atomic Energy Agency, Ibn Tofaïl University, Kenitra, Morocco,; 2 Department of Basic Health Sciences, Deanship of Preparatory Year, Princess Nourah Bint Abdulrahman University, Riyadh, Saudi Arabia,; 3 Direction of Epidemiology and Disease Control, Ministry of Health, Rabat, Morocco,; 4 Nutrition, Non-Communicable Diseases and Mental Health Department, World Health Organization, Regional Office for the Eastern Mediterranean (EMRO), Abdul Razzak Al-Sanhouri, Nasr City, Cairo, Egypt,; 5 Neonatal Medicine and Resuscitation, Pediatrics V, CHIS Ibn Sina, Mother and Child Couple Health and Nutrition Research Team, Faculty of Medicine and Pharmacy, Mohammed V University, Rabat, Morocco

**Keywords:** National strategy, salt reduction, bakers, bread

## Abstract

**Introduction:**

Morocco launched a national salt reduction strategy in 2019. The commitment of bakeries is key in the success of this strategy. However, the evaluation of such a commitment is not yet done. This study aims to examine knowledge of bakers about the national strategy of salt reduction and evaluate their commitment in implementing the specific recommendation related to salt reduction in bread.

**Methods:**

a quantitative exploratory study targeted bakeries (N=432) from all the administrative regions of Morocco. Data was collected using a questionnaire composed of three sections: knowledge of bakers related to national strategy of salt, current contribution of bakers in implementing the national strategy and future commitment towards implementing the national strategy.

**Results:**

about 73% (n=317) bakers lack of knowledge about the recommendations on the progressive reduction of the salt content in bread. Radio and TV were the most used sources to obtain information by bakers (45.2% (n=52) and 35.6% (n=41) respectively). None of the bakers was informed about the process of gradual reduction of salt content in bread, and none of them was committed to it. A total of 60.32% (n=252) of bakeries do not respect the national recommendations of 10g of salt/Kg of flour while 89.6% (n=387) of bakers express their interest in getting committed to the process in the next 2 years.

**Conclusion:**

increasing the knowledge of bakers is highly recommended to guarantee their commitment toward contributing to the strategy of reducing salt in bread. Dissemination of messages via TV and radio could be appropriate.

## Introduction

Even though, the actual salt intake worldwide is more than twice of WHO recommendations (1g/day) with an average of 5 g/day, and ranging between 9-12 g/day [1]. A sodium intake above the recommended level is the primary cause of elevated blood pressure with a global estimated prevalence of 26.4% [2]. In response to this global trend, WHO developed the global action plan targeting a 30% reduction of the average salt intake by 2025 [3]. Consequently, several countries such as Morocco have established voluntary or legislative programs to promote a progressive salt reduction [4].

In Morocco, bread is recognized as the most popular consumed food. The average consumption was estimated at about 500 g/day/individual [5] and the average quantity of salt added during the preparation of white bread is 17.42 grams/kg of wheat flour, being equivalent to a daily intake of 8 to 9 g of salt per bread. Of interest, simple calculation indicates that a Moroccan individual obtains his/her daily recommended salt intake by just consuming bread alone [6]. Thus, logic deductive reasoning indicated that bread constituted a potential food item to be subjected to salt reduction because it would lead to remarkable public health benefits. Previous studies showed that a reduction in salt content of up to 29% is acceptable by consumers [4,7-11]. But this level of reduction faced challenges due to the techno-functional and sensory roles of salt in bread [12]. Thus, bakery industries as an important stakeholder in this process are endeavoring to reduce the salt content in their products [9,13].

In Morocco, wheat consumption per capita is estimated at 173 kg annually, which is among the highest in the world (152 kg) [14]. In addition, the bakery industry is a continuously growing market, their sales increased from 827.4 US$ in 2010 to 1074.5 US$ in 2014 [15]. This implies that achievement of the strategy of salt reduction in bread in Morocco depends on a strong commitment of bakers. Thus, this study aimed to first examine knowledge of bakers, as potential stakeholders, about the national strategy of salt reduction and second evaluate their current contribution and future commitment in implementing the specific recommendation related to salt reduction in bread. As such, appropriate recommendations will be developed targeting the Moroccan bakers and increasing their awareness about the crucial role they have to assume toward a successful reduction of salt in bread in Morocco.

## Methods

**Study design and subjects:** this was a quantitative exploratory study targeting bakeries in Morocco. The survey was conducted during 8 months from January to August 2018. Bakeries included in this study were identified in formal databases provided by the national bakery and pastry federation as well as regional investment centers. The initial database consisted of 2000 bakeries from the 12 regions of Morocco. Bakeries with no phone numbers were excluded (n=284). The bakeries included (n=1716) were classified according to their type as being either artisanal bakery or industrial bakeries. By definition, industrial bakeries are those that emphasize on automation and mechanization of bread production, while artisanal bakeries rely less on mechanization but more on the skill and knowledge of the baker.

**Figure 1:**
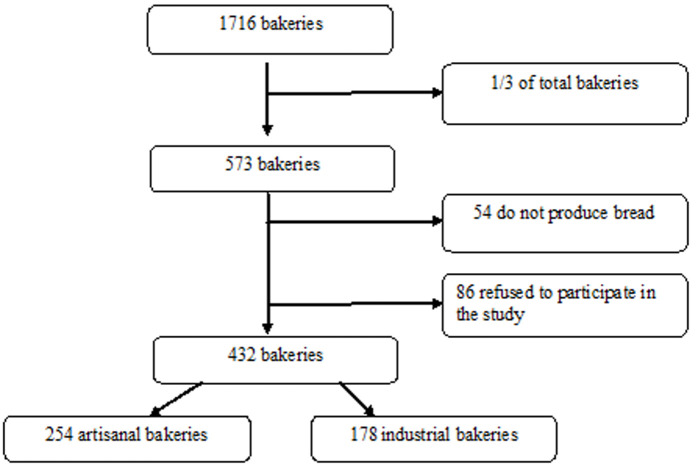
flowchart of the study sample

A sample size of n=572 bakeries was considered for baker´s recruitment to participate in this study. It was obtained using a three-stage sampling strategy. The first stage was to take the 1/3 of all bakeries of each region. The second stage was to subdivide the bakeries by type and the third stage was to realize a simple random sampling to define the bakeries that will be recruited in this study. Out of the calculated sample, 86 bakeries refused to participate because of lack of time or disinterest in taking part in this study and 57 bakeries reported that they do not produce bread locally. A total number of 432 bakeries participated in the survey (Figure 1).

**Research instruments:** data was collected using a questionnaire made up of 3 sections as follows: Section a: it aimed to assess knowledge of bakers related to the national strategy of salt reduction. It included 6 questions, of which 3 were on whether bakers know about the strategy of salt reduction in food and about the specific recommendation of progressive reduction of salt in bread, main source of information related to the national strategy (national bakery and pastry federation, ministry of health, television, radio, internet, newspaper or other sources). The 3 other questions on salt which are: “is salt needed for adequate functioning of the body? (yes/no)”, “does excessive intake of salt have negative health effects? (yes/no)” and “which of the following food items has the highest contribution to salt intake in our diet? (bread, fast-food, salty snacking)”.

Section b: it focused on evaluating the current contribution of bakers in implementing the national strategy of salt reduction in bread. It consisted of 9 questions inquiring whether bakers are aware about the recommendation set by the ministry of health and Moroccan national bakery and pastry federation about the process of gradual reduction of salt content by 10% yearly for 3 years starting from 2015. Being committed to the process of salt reduction, the many grams of salt added to 1 kg of salt wheat before and after the strategy. Taste issues reported by consumers, motivation and barriers toward reducing salt in bread.

Section c: it measured future commitment toward implementing the national strategy of salt reduction by bakers. Two questions were used: would you be committed to the progressive salt reduction in the next 2 years; and what expected barriers would you face due to salt reduction. The questionnaire used in this study was developed based on review of the literature and the questions were modeled on that used in a previous survey (ministry of solidarity and health, 2006). However, some questions were modified to suit better the objective of the study and to contextualize it to Moroccan culture. Questions were also made as simple as possible in order to shorten the duration of the data collection as it was achieved through telephone calls. The questionnaire was translated into Arabic and reviewed by two Arabic speaking nutritionists to ensure that the wording of questions as well as their meaning was correct. A pilot survey was conducted on a sample of 24 bakeries to test the ease of understanding of the questionnaire and to estimate its average timing. Final minor modifications were done and the average duration of the phone calls was 6 min.

**Statistical analysis:** all statistical analyses were performed using SPSS software (version 20.0). Normal distribution of the quantitative variables was checked using Kolmogrov-Smirnov test. Results are presented as frequencies and percentages. The p-values were determined using the chi-square test (the chi-square value was corrected for cells with a theoretical frequency less than 5.

**Ethical considerations:** ethical approval (number 38/15 - 2018) was obtained from the IRB committee of College of Medicine and Pharmacy of Mohammed 5^th^ University (Rabat, Morocco). Data was collected using phone calls and before taking part of the study, bakers were given full explanation of the objective and kind of participation expected from them. An oral consent was given by each of the bakers who agreed to participate in the study. The collected data was maintained private, confidential and anonymous and its use was restricted to research purposes only.

## Results

**Regional distribution of bakeries:** a total sample of 432 bakeries participated in the study, artisanal bakeries representing about 59% (n=254) and industrial bakeries represents 41.02% (n=178). As shown in Table 1, bakeries were taken from the 12 administrative regions of Morocco; highest proportions of bakeries were in the regions of Casablanca-Settat (18.51% (n=80)) and Rabat-Sale-Kenitra (20.83% (n=90)).

**Table 1:** distribution of bakeries according to the 12 administrative regions of Morocco

Regions	All bakeries n (%)	Artisanal bakeries n (%)	Industrial bakeries n (%)
Oriental	40 (9.25)	29 (11.42)	11 (6.18)
Fès-Meknès	30 (6.94)	20 (7.87)	10 (5.62)
Rabat-Salé-Kenitra	90 (20.83)	67 (26.38)	23 (12.92)
Casablanca-Settat	80 (18 .51)	51 (20.08)	29 (16.29)
Béni Mellal-Khénifra	10 (2.31)	1 (0.39)	9 (5.05)
Marrakech-Safi	45 (10.42)	25 (9.84)	20 (11.24)
Draa-Tafilalt	8 (1.85)	3 (1.18)	5 (2.81)
Souss-Massa	40 (9.25)	24 (9.45)	16 (8.99)
Tanger-Tétouan	60 (13.90)	21 (8.27)	39 (21.91)
Guelmim-Oued Noun	10 (2.31)	7 (2.76)	3 (1.68)
Laayoune-Sakia el Hamra	15 (3.50)	4 (1.57)	11 (6.18)
Dakhla Oueddahab	4 (0.93)	2 (0.79)	2 (1.12)
Total	432 (100)	254 (100)	178 (100)

**Knowledge of bakers related to national strategy of salt reduction:** in Table 2 are presented the information about baker´s knowledge related to the national strategy of salt reduction. 26.6% (n=115) of bakers have heard about actions led by the ministry of health to reduce salt content in the diet and bread. No statistically significant difference (p>0.05) was obtained between the two types of bakeries. Radio was the most used source of information with 45.2% (n=52) followed by television 35.6% (n=41). All bakers (100%) confirmed that salt isn´t essential for the adequate functioning of the body and that excessive intake leads to a negative effect on health. Regarding food items with the highest contribution to salt intake in the diet, 60.2% (n=260) of bakers reported that it is the fast foods, this percentage was higher in artisanal compared to industrial bakeries (63.8% (n=162) versus 55.1% (n=98), respectively) (p <0.05).

**Table 2:** knowledge related to the national strategy of salt reduction

		Artisanal bakeries	Industrial bakeries	
	Total N (%)	N (%)	N (%)	p-value
**Do you know about the national strategy of salt reduction by the ministry of health?**				
Yes	115 (26.6)	66 (26.0)	49 (27.5)	0.721
No	317 (73.4)	188 (74.0)	129 (72.5)	
**Do you know about the specific recommendation of progressive reduction of salt in bread by the ministry of health?**				
Yes	115 (26.6)	66 (26.0)	49 (27.5)	0.721
No	317 (73.4)	188 (74.0)	129 (72.5)	
**Source of information related to the national strategy of salt reduction national bakery and pastry federation**				
Yes	6 (5.2)	5 (7.6)	1 (2.0)	0.187
No	109 (94.8)	61 (92.4)	48 (98)	
**Ministry of health**				
Yes	0 (0)	0 (0)	0 (0)	N/A
No	115 (100.0)	66 (100.0)	49 (100.0)	
**Television**				
Yes	41 (35.6)	24 (36.8)	17 (34.7)	0.986
No	74 (64.3)	42 (63.64	32 (65.3)	
**Radio**				
Yes	52 (45.2)	29 (43.9)	23 (46.9)	0.630
No	63 (54.8)	37 (56.1)	26 (53.1)	
**Internet**				
Yes	16 (13.9)	8 (12.12)	8 (16.3)	0.519
No	99 (86.1)	58 (87.88)	41 (83.7)	
**Newspapers**				
Yes	0 (0.0)	0 (0.0)	0 (0.0)	
No	115 (100.0)	66 (100.0)	49 (100.0)	N/A
**Is salt needed for adequate functioning of the body?**				
Yes	0 (0.0)	0 (0.0)	0 (0.0)	
No	432 (100.0)	254 (100.0)	178 (100.0)	N/A
**Excessive intake of salt has negative health effects?**				
Yes	432 (100.0)	254 (100.0)	178 (100.0)	
No	0 (0.0)	0 (0.0)	0 (0.0)	N/A
**The food items with highest contribution to salt intake in our diet**				
Bread	36 (8.3)	14 (5.5)	22 (12.4)	
Fast-food	260 (60.2)	162 (63.8)	98 (55.1)	0.026
Salty snacking	136 (31.5)	78 (30.7)	58 (32.6)	

The p-values were determined using the Chi2 test. The value was corrected for cells with a theoretical frequency <5

**Figure 2:**
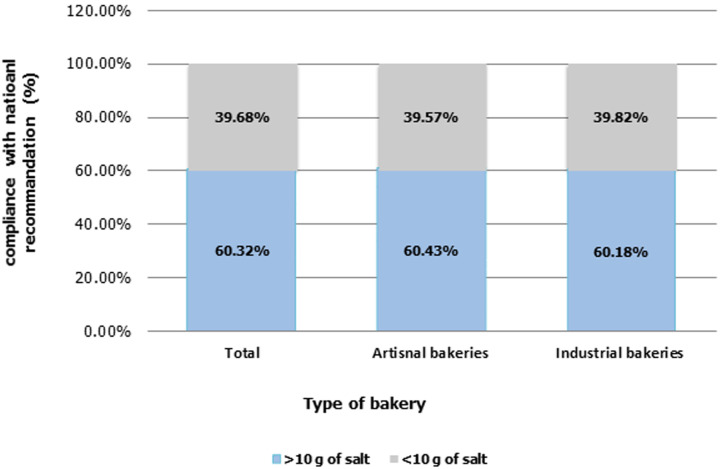
compliance with the national recommendations for the salt content in bread (10g/Kg of flour)

**Current contribution of bakers in implementing the national strategy:** results regarding the current contribution of bakers in implementing the national strategy of salt reduction are presented in Table 3. All the bakers (100%) interviewed were not informed about the process of gradual reduction of salt content in bread and therefore none of them was committed to this process. Thus, no answers were obtained for the last four subsequent questions. Regarding the regulation of the amount of salt in bread, 87.7% (n=39.7) of bakers have proposed that the same dose of salt should be regulated by law in all bakeries. Compliance with national recommendations for the salt content in bread (10g/Kg of flour) is shown in the figure. A total of 60.32% (n=152) of bakeries do not respect the national recommendations of 10g of salt/Kg of flour, and this for the 2 types of bakeries (Figure 2).

**Table 3:** current contribution of bakers in implementing the national strategy of salt reduction

	Total n (%)	Artisanal bakeries n (%)	Industrial bakeries n (%)	p-value
**In your opinion, should you regulate the dosage of salt in bread?**				
Yes	379 (87.7)	218 (85.8)	161 (90.4)	0.149
No	53 (12.3)	36 (14.2)	17 (9.6)	
**Have you been informed of the process of progressive reduction of salt content in bread at the rate of 10% per year for 3 years since 2015?**				
Yes	0 (0)	0 (0)	0 (0)	N/A
No	432 (100.0)	254 (100.0)	178 (100.0)	N/A
**Being committed to the process of salt reduction?**				
Yes	0 (0)	0 (0)	0 (0)	N/A
No	432 (100.0)	254 (100.0)	178 (100.0)	N/A
What motivate you to reduce the salt content in bread?*				
Were any taste issues reported by consumers?*				
What were the main barriers toward reducing the salt content in bread?*				
What obstacles do you encounter in bakery to reduce the salt in bread?*				

* No answers were obtained because no bakery is committed in the salt reduction process; the p-values were determined using the Chi2 test (the chi-square value was corrected for cells with a theoretical frequency <5

**Future commitment towards implementing the national strategy:** Table 4 summarizes future commitment of bakers towards implementing the national strategy; in which, 89.6% (n=387) have shown interest in joining the process in the next 2 years, with no significant difference between the two types of industries. According to the bakers, the loss of consumers seems to be the main obstacle of reducing the salt content in bread.

**Table 4:** future commitment of bakers in implementing the national strategy of salt reduction

	Total n (%)	Artisanal bakeries n (%)	Industrial bakeries n (%)	p-value
**Do you think you can commit to gradually reducing salt in bread over the next 2 years**				
Yes	387 (89.6)	222 (87.4)	165 (92.7)	0.076
No	45 (10.4)	32 (12.6)	13 (7.3)	
**What difficulties would you be afraid of encountering in reducing the salt content in bread**				
No barrier	401 (92.8)	226 (89.0)	175 (98.3)	0.770
Loss of consumer	31 (7.2)	28 (11.0)	3 (1.7)	

The p-values were determined using the Chi2 test (the chi-square value was corrected for cells with a theoretical frequency <5)

## Discussion

This study aimed to explore the baker´s perspective with regard to their contribution to the implementation of the national strategy of salt reduction in Morocco. Our results revealed the limited knowledge of the bakers about the national strategy led by the ministry of health. It also indicated that none of the bakers who participated in this study (from both types artisanal and industrial) was informed about the process of gradual reduction of salt content in bread and therefore none of them was committed to this process. As a consequence, in terms of practice, more than half of bakeries do not respect the national recommendations of 10g of salt/kg of flour. Hence, most bakers express their interest in getting committed to the process in the next 2 years, although the possible loss of customers was considered as an obstacle which could affect the serious intention in engaging in the process.

Developing and implementing a strategy can be a complex and difficult process; it becomes even more challenging when the stakeholders are insufficiently engaged. It is well demonstrated that within the context of any strategy implementation, commitment and involvement of different stakeholders is determinant to its success and goals achievement. In the case of the present study, one of the main stakeholders is bakeries. In fact, contribution of processed food to salt consumption ranges between 70 to 75%. In addition, bread consumption could be considered as a major contributor to the high amounts of salt intake by Moroccan. According to our study, the average amount of salt added to the bread is 12.5g/kg. Taking into consideration its consumption of 500 g/day/person [5], the bread consumption alone delivers 6.25 g of salt per day, exceeding the WHO recommendations for daily salt intake (5g/day). A study carried out in eight countries of the eastern Mediterranean region showed that the average bread salt ranged from 4.28 g/kg in Jordan to 12.41 g/kg in Tunisia. This contributes to a daily salt intake ranging from 1.3 g (12.5% of daily salt intake) in Jordan to 3.7g (33.5% of daily salt intake) in Tunisia [16]. These facts clearly indicate that bakers should be involved through a participatory approach to contribute efficiently in the salt reduction strategy.

The national bakery and pastry federation as an organized body with 1716 members could play a key role in promoting the implementation of the strategy and sharing all related information. In fact, our results indicate that their general knowledge is poor and null as per the specific progressive reduction of salt in bread. Because of this situation, it is logical to expect the unhealthy practice by the majority of backers as per adding an amount of salt higher than 10 g/kg of flour (recommended by the ministry of health). According to our study radio and TV were identified as the most used sources to obtain information by bakers. Therefore, they could be considered a good channel disseminating information related to the strategy as evidence-based from previous studies [17]. This could be a feasible approach to enhance their knowledge through tailored messages leading to an increase of bakers´ motivation to implement salt reduction in bread. In fact, in France a study concluded that the information on salt reduction recommendations seemed to be sufficient for half of artisan bakers and hypermarkets and sufficient for 70% of the industrial bakeries [18].

Within the projection of future contribution in implementing the strategy, our findings revealed that 87.4% (n=222) and 92.7% (n=165) of artisanal and industrial bakers, respectively, confirmed their interest in committing to the progressive salt reduction in bread. However, and in disagreement with previous studies, they indicated that the susceptibility of taste alteration and the consequent loss of customers are not foreseen as potential factor that could hinder their commitment of salt in bread [19,20]. There is no doubt that satisfying customers´ taste is the primary concern of the bakeries. Still, a two-sided education intervention would also be appropriate in this case. As on one hand, it needs to strengthen bakers´ belief that a reduction in salt content is acceptable by consumers. In fact, several studies confirmed that bread with less quantity of salt than usual was almost undetected compared to regular bread even after a significant reduction in salt [7-10]. Besides, according to findings published recently by Guennoun *et al*. 2019, 21.8% of the Moroccan consumers agree to purchase bread with 23% salt reduction and 41.8% agree purchasing bread at 16% of salt reduction, whereas 20.4% at 30% of salt reduction [11]. In addition, La Croix *et al*. 2010, reported that reducing sodium levels in bread up to 30% did not affect consumer liking or purchase intent of the products [21]. On the other hand, educating consumers should be given equal importance as bakers. As such, it is demonstrated that increasing awareness about salt recommendations generates a form of public accountability and consequently, a demand of customers with less salted bread and better acceptance. Therefore, informing consumers remains essential in so far as bakers declare themselves ready to adapt their habits to customer demand.

The present study is very informative and to our knowledge it is the first research carried out in Morocco in order to assess the knowledge of bakers, their current contribution and the future commitment towards implementing the national strategy. However, the main limitation of this study is that it targeted only bakeries members of the national bakery and pastry federation of Morocco and regional investment center, while the informal sector was not included knowing that it controls more than 50% of the market, as confirmed by the president of the national federation of bakery and pastry (personal communication). Yet, the sampling technique used in this study allowed a good representativeness for the different regions in Morocco and in most of the obtained results no significant differences were obtained between artisanal and industrial bakeries. The other limitation to be considered in this study is the phone calls as a technique for data collection. Though, it was tested and found to be effective. However, in a few cases, obtaining the complete information was challenging.

## Conclusion

This study shows that backers lack knowledge about the national strategy of salt reduction in bread. Bakers show willingness to engage in implementing the gradual reduction of salt in bread. Hence the need to increase knowledge and awareness of bakers by adopting appropriate strategies to the dissemination of messages, by involving the TV and radio in view of their major influence on bakers. In addition, the role of the national bakery and pastry federation should be promoted as an organized body representing bakeries in Morocco. Communication strategies should also target consumers as it would support further the successful achievement of the strategy.

### What is known about this topic

- WHO developed the global action plan targeting a 30% reduction of the average salt intake by 2025;
- Strategy of reducing salt in bread in Morocco is conceived to reduce prevalence of non-communicable diseases;
- The consumption of salt in Morocco exceeds the recommendations of the World Health Organization.

### What this study adds

- First study at national level aiming to reduce salt consumption in food;
- Backers lack knowledge about the national strategy of salt reduction in bread;
- The awareness of bakers and consumers is a key element for the success of the national strategy of salt reduction in Morocco by involving the TV and radio in view of their major influence on bakers.
